# Quantitative Segmentation Analysis of the Radiological Changes by Using ITK-SNAP: Risk Assessment of the Severity and Recurrence of Medication-related Osteonecrosis of the Jaw

**DOI:** 10.7150/ijms.56408

**Published:** 2021-03-24

**Authors:** Yu-qiong Zhou, Gee-hun Son, Yue-qi Shi, Ye-jia Yu, Meng-yu Li, Qin Zhang, Duo-hong Zou, Zhi-yuan Zhang, Chi Yang, Shao-yi Wang

**Affiliations:** Shanghai Ninth People's Hospital, Collage of Stomatology, Shanghai Jiao Tong University School of Medicine (200011); National Clinical Research Center of Oral Disease; Shanghai Key Laboratory of Stomatology & Shanghai Research Institute of Stomatology, Department of Oral Surgery.

**Keywords:** medication-related osteonecrosis of the jaw, quantitative radiographic analysis, ITK-SNAP, recurrence

## Abstract

**Background and purpose:** Medication-related osteonecrosis of the jaw (MRONJ) severely impairs patients' quality of life and is remarkably refractory to treatment. There are lots of studies about identification of the radiographic features of MRONJ, yet reports about quantitative radiographic analysis for the risk assessment of the severity and recurrence of MRONJ are rarely heard. The aim of this study was to investigate the volumes of osteolytic lesions and radiodensity values of osteosclerotic lesions in MRONJ patients by using ITK-SNAP for severity prediction and prognosis evaluation.

**Materials and methods:** Of 78 MRONJ patients (78 lesions) involved in this retrospective study, 53 were presented as osteolytic lesions and 25 were presented as osteosclerotic changes alone. Comprehensive CBCT images, demographics and clinical data of patients were investigated. The volumetric analysis and radiodensity measurement were performed by ITK-SNAP. SPSS 25.0 were used for statistical analysis.

**Results:** The osteolytic lesion volumes in MRONJ patients receiving intravenous bisphosphonates (P=0.004) and patients without osteoporosis (P=0.027) were significantly large. No significant correlation between the volumes and bisphosphonates duration was found (P=0.094). The radiodensity values of osteosclerotic lesions was significantly correlated with bisphosphonates duration (P=0.040). The surrounding area of post-surgical lesions in MRONJ patients with recurrence showed significantly great radiodensity values (P=0.025). No significant correlation between the radiodensity values and the transformation from osteosclerotic lesions to osteolytic lesions was observed (P=0.507).

**Conclusion:** MRONJ patients receiving intravenous bisphosphonates develop into large volumes of osteolytic lesions more easily. Long-term bisphosphonates duration is possibly related with higher bone density of osteosclerotic lesions, while higher density is not associated with the transformation from osteosclerotic lesions to osteolytic lesions. A rise of bone mineral density nearby post-surgical lesions is probably a predictor for MRONJ recurrence.

## Introduction

Medication-related osteonecrosis of the jaw (MRONJ) is a serious disease that severely impairs patients' quality of life. The use of bisphosphonates, denosumab 1 and some antiangiogenic drugs 2 are considered to be related with MRONJ. The radiological changes of MRONJ are distinct, including osteolysis, sequestrum, trabecular sclerosis, cortical erosion, periosteal reaction and residual tooth sockets et al. 3, 4. Osteosclerosis is possibly an early radiographic manifestation of MRONJ 5-8. This is induced by the toxicity of bisphosphonates on osteoclasts, which disturbing the function and accelerating apoptosis of osteoclasts, leading to the decrease of bone turnover 9. Osteolysis and sequestrum are the most common radiographic features of MRONJ. It may be related with the local vascular insufficiency and bacterial infection of the jaw 10.

Different radiographic features are not self-existent. Osteosclerosis appears to be the precursor of osteolysis or sequestrum 5, 11, 12. Shin et al. reported that a mix of osteolysis and sclerosis was the most common manifestation on panoramic radiographs 13. Sequestrum often co-exists with osteolysis, forming different volumes of osteolytic lesions. To some extent, the volumes of lesions and the alveolar bone mineral density are connected with the clinical stage of MRONJ, or clinical severity in other words 14, 15. The volumetric and radiodensity analysis could be of value for prediction and prevention of MRONJ.

Cone-beam computed tomography (CBCT) can provide precise evaluation of osseous changes including volumetric analysis 16 and radiodensity measurement 17. ITK-SNAP, as an open-source software, is available for quantitative radiographic analysis. Vallaeys et al. 18 firstly used ITK-SNAP in analysis of dento-maxillary osteolytic lesions. They demonstrated that manual segmentation in ITK-SNAP was more reliable for analysis of osteolytic lesions than semi-automatic segmentation. Based on this result, Zirk et al. 16 investigated the osteolysis volumes by ITK-SNAP with combination of manual segmentation and semi-automatic segmentation, and analyzed the association of osteolysis volumes with gender, age and the affected jaw. Their results revealed significantly greater volumes of osteolytic lesions in males.

MRONJ is notably refractory to the treatment which includes conservative approaches and invasive surgical interventions 19-21. The separated sequestrum is beneficial to an effective surgery for its viable border. However, osteosclerotic lesions have no well-defined boundary, which usually results in excessive surgical damage to the jawbone or incomplete surgery leading to recurrence. Thus, the transformation from osteosclerotic lesions to a well-defined sequestrum (osteolytic lesions) is meaningful for the surgery.

In the present study, we focused on two trabecular changes in cancellous bone, classified the MRONJ lesions into two types including osteolytic lesions and osteosclerotic lesions, and investigated the predictors for volumes and radiodensity of two lesions respectively. Additionally, the potential associations between the transformation from osteosclerotic lesions to osteolytic lesions or post-surgical recurrence and radiodensity were evaluated. To our knowledge, this is the first study researching CBCT images of MRONJ with combination of volumes and radiodensity measurement by ITK-SNAP via segmentation analysis.

## Materials and methods

### Inclusion and exclusion criteria

Between January 2016 and January 2019, 78 patients receiving bisphosphonates and diagnosed with medication-related osteonecrosis of the jaw (MRONJ) were involved in this study. All patients were diagnosed by professional clinicians in the Department of Oral Surgery at the Ninth People's Hospital according to the 2019 clinical practice guideline of MRONJ 14. The diagnosis standard was as follows: (1) current or previous treatment with a BMA or angiogenic inhibitor, (2) exposed bone or bone that can be probed through an intraoral or extraoral fistula in the maxillofacial region and that has persisted for longer than 8 weeks, and (3) no history of radiation therapy to the jaws or metastatic disease to the jaws. Those with developmental maxillofacial anomalies, previous radiation in the head and neck area and jaw bone tumors were excluded from the study.

This study was conducted with the understanding and written consent of every patient and in accordance with the tenets of the Declaration of Helsinki. This study was independently reviewed and received approval from the institutional ethical committee of the Ninth People's Hospital of Shanghai Jiao Tong University School of Medicine (SH9H-2020-T37-3).

### Variables and cone beam computed tomography (CBCT) images collection

All variables and data were collected by the same researcher who was blind to the study method. The demographics and clinical features, including gender, age, bisphosphonates duration and routes, affected jaws, osteoporosis, chemotherapy use, targeted drugs use and immune suppressive drugs use, were recorded.

All patients were followed over a year with the examination of CBCT. Sufficient CBCT images were required for all involved patients. For the patients receiving surgery, the initial, pre-surgical and post-surgical CBCT images were collected. CBCT scans were taken by the same technician using the same device (Planmeca Oy, Helsinki, Finland; tube voltage: 90 kV; tube current: 11-13 mA; exposure time: 12 s) in the Oral Radiology Department at the Ninth People's Hospital. The field of view was 13 × 5.5 cm and the voxel size was 200 μm. The CBCT images of 40 healthy people who had no jaw bone anomalies were investigated and measured as the control of radiodensity values. Age and sex had been demonstrated no statistical differences between the patients and the control.

**Figure 1:**
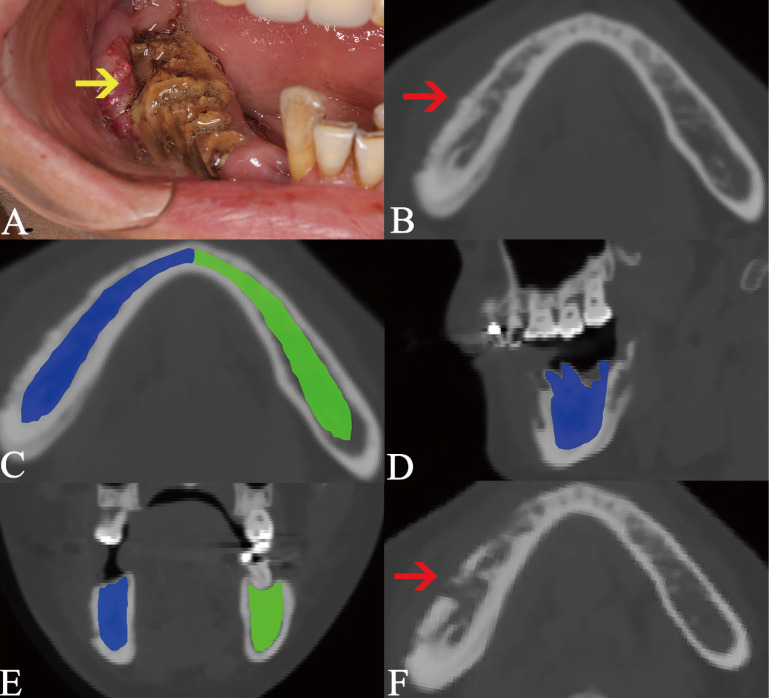
Osteosclerotic lesion and its transformation to osteolytic lesion. The lesion is pointed out by the arrow in intraoral photograph (A) and axial plane (B). Due to the lesion was located at two areas including A_R_ and B_R_, the segmentation boundary was set from the mandibular symphysis to the anterior of ramus (C, D, E). The segmentation of cancellous bone in sclerotic lesion is colored as blue, while the same area in the bilateral side is segmented as the control and colored as green. The transformation from osteosclerotic lesion to osteolytic lesion is presented (F).

**Figure 2:**
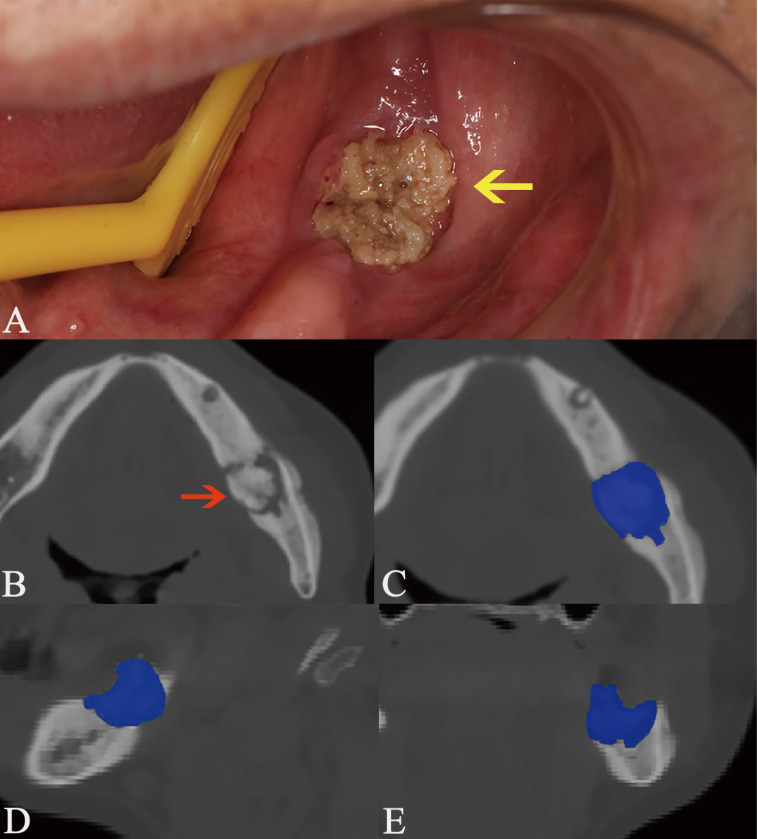
Osteolytic lesion containing sequestrum. The lesion is pointed out by the arrow in intraoral photograph (A) and axial plane (B). The manual segmentation of CBCT images in axial (C), sagittal (D), and coronal plane (E) is highlighted as blue area.

**Figure 3:**
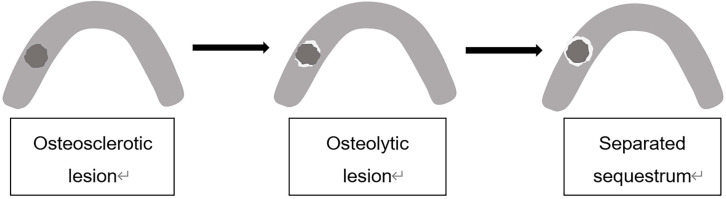
The defined MRONJ lesions in radiography. The osteosclerotic lesion is presented as deep grey which signifies its higher radiodensity than the jaw. Osteolysis is indicated by white color which signifies lower radiodensity.

In this study, we focused on the trabecular changes in cancellous bone and classified the MRONJ lesions into two types, including osteosclerotic and osteolytic lesions (Fig. 1A, B; Fig. 2A, B). We defined osteosclerotic lesions as the increased alveolar bone density without any other trabecular changes. Once osteolysis occurs, the radiographic type of MRONJ lesions was classified as osteolytic lesions. The osteosclerotic lesion only presented as sclerosis in CT imaging, while the osteolytic lesion could involve many CT imaging features, such as osteolysis, sequestrum and sclerosis. The defined MRONJ lesions in radiography were depicted in Figure 3.

In this study, the transformation from osteosclerotic lesions into osteolytic lesions was do observed in many MRONJ patient. Among those patients, we measured the radiodensity values of osteosclerosis which was the initial manifestation in radiography and recorded the transformation from osteosclerotic lesions to osteolytic lesions. Additionally, post-surgical recurrences and the radiodensity values around the post-surgical lesions were recorded as well.

### Volumetric analysis and radiodensity values measurement

**Figure 4:**
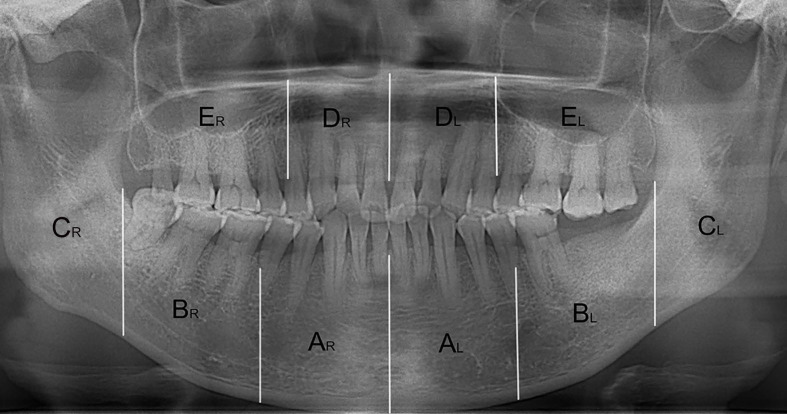
The resected regions in mandible and maxilla. A_R_/ A_L_: from the mandibular symphysis to the mental foramen; B_R_/ B_L_: from the mental foramen to the anterior of ramus; C_R_/ C_L_: the mandibular ramus. D_R_/ D_L_: from the incisive foramen to the medial wall of maxillary sinus; E_R_/ E_L_: the residual alveolar bone.

**Figure 5:**
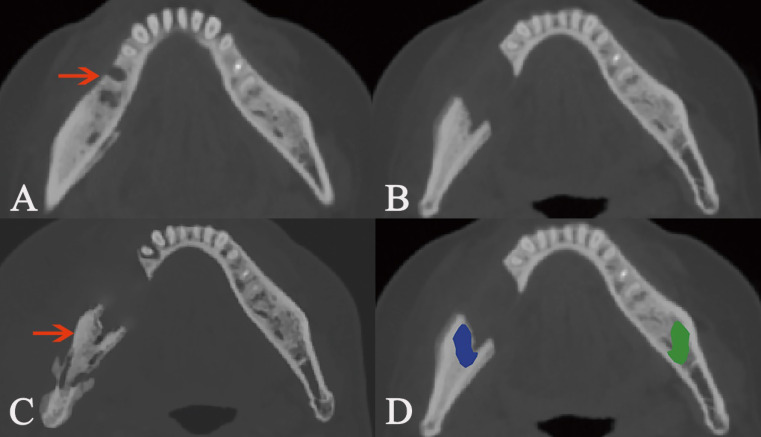
The recurrence of post-surgical lesion. Red arrow points out the pre-surgical MRONJ lesion (A). Image B shows the lesion after surgery. Yellow arrow indicates the recurrence of post-surgical lesion (C). The segmentation of cancellous bone nearby post-surgical lesion is colored as blue, while the same area in the bilateral side is segmented as the control and colored as green (D).

The open-source software ITK-SNAP (Penn Image Computing and Science Laboratory) was used to measure the volumes of osteolytic lesions and calculate the mean radiodensity values of osteosclerotic lesions. The volumes and radiodensity measurement were realized by manual segmentation. The detailed manual segmentation process was as follows:

- All CBCT images were imported as DICOM datasets and showed slices of three dimension;
- Two modes, including polygon and paintbrush, can be used for manual segmentation. The first was performed by drawing and filling polygons in the three orthogonal image slices. The other was performed by paintbrush-like tool. Polygon mode was used to segment the boundary of lesions initially, while the paintbrush mode was used to improve and complete the segmentation.
- For the osteosclerotic lesion, we resected the mandible and maxilla in ten regions and measured the radiodensity values of relevant regions which were comprehensively decided by the location of intraoral exposed bone and symptomatic areas (Fig. 1). The resected regions are stated as follows and showed in Figure 4. All CBCT slices in three views of a lesion were segmented according to the boundary of relevant regions and the average radiodensity values were calculated. (Mandible, A_R_/ A_L_: from the mandibular symphysis to the mental foramen; B_R_/ B_L_: from the mental foramen to the anterior of ramus; C_R_/ C_L_: the mandibular ramus. Maxilla, D_R_/ D_L_: from the incisive foramen to the medial wall of maxillary sinus; E_R_/ E_L_: the residual alveolar bone).
- As for the osteolytic lesion, the region was segmented along with the periphery of osteolysis (Fig. 2). All CBCT slices in three views of a lesion were segmented and the volume was calculated.
- The radiodensity measurement of the surrouding of post-surgical lesion was revealed in Figure 5. The segmented boundary was approximately 1 cm diameter away from the post-surgical lesion and the average radiodensity value of the surrounding of post-surgical lesion was calculated.
- To eliminate the individual differences, two radiodensity indexes were calculated by compared the mean radiodensity values with the contralateral non-affected area at the same jaw (ratio 1) and with the same location in healthy people (ratio 2). For example, if a lesion belongs to the region B_R_ intraorally, the radiodensity values of the region B_R_ are calculated, then the radiodensity values of the region B_L_ and the region B_R_ in health people are recorded as control. Additionally, if the lesion is located on the two anterior region such as region A_R_ and A_L_ or D_R_ and D_L_, the mean radiodensity values of two posterior regions (B_R_ and B_L_; E_R_ and E_L_) and the region A_R_ and A_L_ or D_R_ and D_L_ in healthy people are considered as control.

Three specialized radiologists were responsible for the manual segmentation analysis. The average was considered as the final result for enhancing the reliability.

### Statistical analysis

SPSS 25.0 (SPSS Inc, Chicago, IL) was used for statistical analysis. We calculated the intraclass correlation efficient to ensure the low inter-observer differences. After checking the normal distribution via the Shapiro-Wilk test, the average and standard deviation of continuous variables were calculated. The correlations between volumes or radiodensity values and continuous variables, including age or bisphosphonates duration, were defined by general linear regression. Categorical variables, such as gender, location, osteoporosis, administrated routes of bisphosphonates and medication use, were reported as number or percentage of patients with the characteristic of interest. Independent-sample t test was used to evaluate the correlations between volumes or radiodensity values and categorical variables. The statistical difference of radiodensity values between groups were estimated by independent-sample t test as well. Probabilities of less than 0.05 were accepted as significant.

## Results

Out of the 78 patients (78 lesions) studied, 37 (47.4%) were male and 41 (52.6%) were female. 66 (84.6%) patients had received intravenous bisphosphonates and 12 (15.4%) patients had been treated with bisphosphonates orally. The MRONJ lesions were classified as osteolytic lesions and osteosclerotic lesions by CBCT images. 53 MRONJ lesions were presented as the former and 25 were presented as the latter at the first visit. The intraclass correlation efficient was 0.924, which demonstrated the low inter-observer differences.

The correlation assessment of osteolytic lesion volumes in MRONJ patients and different variables is recorded in Table 1. In 53 patients, the average osteolytic lesion volumes in total were 3920.20 mm^3^. The osteolytic lesion volumes among patients receiving intranvenous bisphosphonates were significantly larger than that among patients with administration of oral bisphosphonates (P=0.004). Similarily, there was a significant difference in osteolytic lesion volumes between patients with osteoporosis versus patients without osteoporosis (P=0.027). No statistical differences were detected in osteolytic lesion volumes based on bisphosphonates duration, gender, affected jaws, chemotherapy, targeted drugs and immune suppressive drugs.

The average radiodensity values of 25 osteosclerotic lesions were 896.25 (Table 2). The correlations of radiodensity values and bisphosphonates duration, gender, affected jaws, chemotherapy, targeted drugs and immune suppressive drugs were estimated statistically. Bisphosphonates duration was found to be significantly related with the radiodensity values of osteosclerotic lesions (P=0.040). The linear regression equation was BMD=785.281+(3.246× bisphosphonates duration). Howerver, the adjusted R^2^ was only 0.217. Other variables showed no significant correlation with radiodensity values of osteosclerotic lesions.

**Figure 6:**
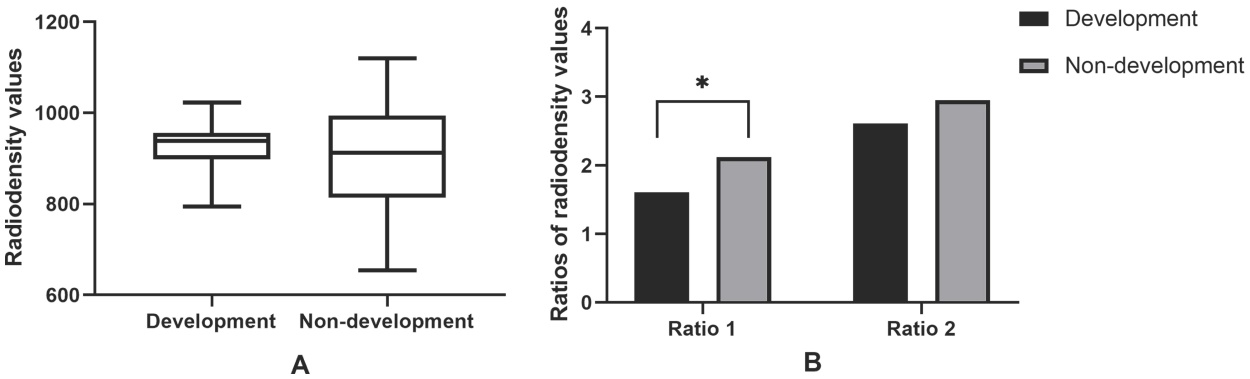
Radiodensity analysis in osteosclerotic MRONJ lesions. 10 osteosclerotic lesions developed into sequestrum, while 15 had no transformation from osteosclerotic lesions to osteolytic lesions. Ratio 1 means the relative value of radiodensity in osteosclerotic lesions and the contralateral side on the same jaw. Ratio 2 means the relative value of radiodensity in osteosclerotic lesions and the same location of healthy people. *P values were statistically significant. Note that only ratio 1 of transformation group was significantly higher than the non-transformation (Figure 6B).

Of 25 osteosclerotic lesions, 10 were developed into osteolytic lesions and 15 were not. The average radiodensity values in maxilla and mandible of healthy people were 290.17 and 450.93, respectively. Two relatively ratios (ratio 1 and ratio 2) were calculated. The average ratio 1 was 1.92, and the average ratio 2 was 2.81 (Figure 6). Ratio 1 in the transformation group was significantly higher than in the non-transformation (P=0.041). No statistically significant differences in radiodensity values and ratio 2 were found between two groups (P=0.507; P=0.195).

**Figure 7:**
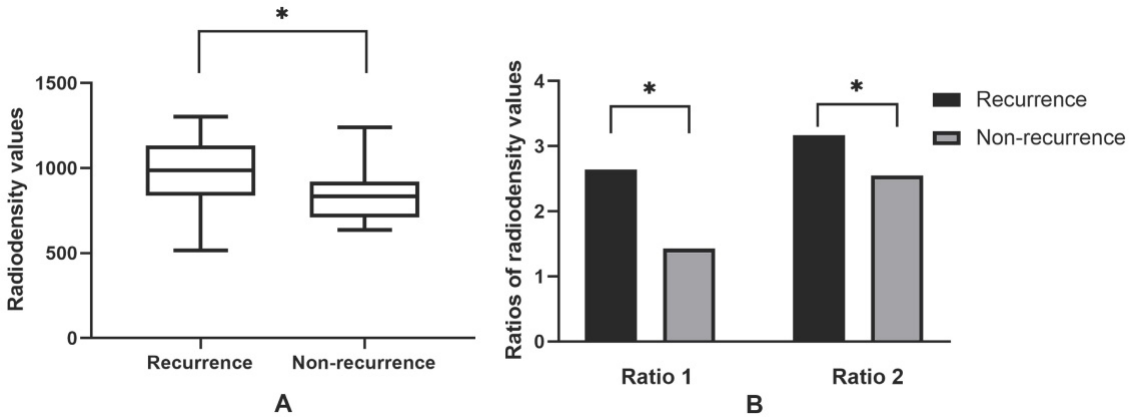
Radiodensity analysis in the surrounding of post-surgical MRONJ lesions. 16 MRONJ lesions occurred recurrences after surgery (n=16), while 25 post-surgical lesions had no recurrences. Ratio 1 means the relative value of radiodensity in the surrounding areas of post-surgical lesions and the contralateral side on the same jaw. Ratio 2 means the relative value of radiodensity in the surrounding areas of post-surgical lesions and the same jaw of healthy people; *P values mean statistically significant. Note that the average radiodensity values (Figure 7A), ratio 1 and ratio 2 (Figure 7B) of recurrence group were significantly higher than the non-recurrence.

41 patients had been performed with surgery and 16 patients were recurred after surgery. Of 16 recurrences, 13 occurred in the patients receiving sequestrectomy and 3 were found in the patients receiving block resection of necrotic bone. In 41 patients, the average radiodensity values were 888.03. The average ratio 1 was 1.90, and the average ratio 2 was 2.80 (Figure 7). The recurrence group showed significantly greater radiodensity values (P=0.025), ratio 1 (P=0.029) and ratio 2 (P=0.026) than the non-recurrence.

## Discussion

In the present study, we defined and mainly investigated two radiographic MRONJ lesions - osteosclerotic and osteolytic lesions. The osteosclerotic lesion only presented as sclerosis in CT imaging, while the osteolytic lesion could involve many CT imaging features, such as osteolysis, sequestrum and sclerosis. Quantitative radiographic analysis was used to analyze the volumes of osteolytic MRONJ lesions, measure the radiodensity values of osteosclerotic lesions and investigate the correlations of transformation from osteosclerotic lesions to osteolytic lesions, post-surgical recurrence and radiodensity values. To our knowledge, this is the first study predicting the severity and recurrence of MRONJ by analyzing both volumes and radiodensity of MRONJ lesions.

Oral bisphosphonates are prescribed for osteoporosis and paget's disease treatment, while intranvenous (IV) bisphosphonates are used primarily for the treatment of cancer-related conditions to reduce skeletal complications 22. MRONJ occurs more frequently in patients administered with IV bisphosphonates rather than oral bisphosphonates 23. The time to MRONJ onset of patients receiving IV bisphosphonates are ususally shorter than oral bisphosphonates 24. However, MRONJ patients with administration of IV bisphosphonates showed a significantly larger osteolytic lesion volumes than oral bisphosphonates in this study. This is more likely because that IV bisphosphonates have a faster accumulation in the bone resulting in a more rapid and insidious bone turnover suppression 25, 26. Additionally, patients with IV bisphosphonates administration ususally have bone marrow and immue suppression by chemotherapy and targeted drugs. Similarily, MRONJ patients without osteoporosis had demonstrated a significantly larger osteolytic lesions volumes than those with osteoporosis. This is because most of MRONJ patients with osteoporosis have received oral bisphosphonates as regular treatment so that smaller lesions are presented.

We found bisphosphonates duration was related with the radiodensity values of osteosclerotic lesions in this study. However, the adjusted R^2^ was only 0.217, indicating that the correlation degree was low. Thus, we need to be cautious in concluding that the radiodensity values of osteosclerotic lesions are influenced by bisphosphonates duration. Additionally, no significant correlation of bisphosphonates duration and osteolytic lesion volumes was found. It may be considered that the formation of osteolytic lesions is multifactorial and exists individual difference. Robert et al. 25 reported that the size of the exposed bone, which measured by oral examination, was directly related with bisphosphonates duration. However, the outcomes was limited due to the small samples and no sufficient statistical envidence.

Chemotherapy, targeted drugs and corcosteroid are considered to increase the risk for developing MRONJ 27-30. Bi et al. 27 found that combined administration of bisphosphonates and chemetherapy caused larger areas of dead bone and soft tissue defects. Zirk et al. 16 reported that males showed greater osteolytic lesion volumes than females. However, no correlation was found between volumes of osteolytic lesions, radiodensity values of osteosclerotic lesions and those factors in this study. Further prospective studies are necessary to provide more scientific evidence.

For osteosclerotic MRONJ lesions, the surgury border are hard to identify, which easily cause excessively surgery or post-surgical recurrence. A well-defined sequestrum is more beneficial to identify appropriate surgery border than osteosclerotic lesions. Thus, the transformation from osteosclerotic lesions to osteolytic lesions is meaningful for clinicians. However, the radiodensity values of osteosclerotic lesions seem not to be related with the transformation in the present study. Whether radiodensity values can indicate the transformation from osteosclerotic lesions into osteolytic lesions, still needs to be studied prospectively.

Our study demostrated that the radiodensity values of the surrouding of post-surgical lesions in patients with recurrence were absolutely and relatively higher than those patients without recurrence. It would be clinically useful to alert the clinician of an increased risk of recurrence if the radiodensity values around the post-surgical lesions are abnormally high. Takaishi et al. 31 also reported that a rise of alveolar bone mineral density occurred near the necrotic lesion of MRONJ, which suggested a tendency of new MRONJ onset.

This study is blinded that researchers in charge of radiographic measurement had unconscious of the grouping and patients' clinical information. However, there still exsit some limitations. The most noteworthy of which was that only manual segmentation was used for radiographic measurement. Although manual segmentation is the relatively precise method, it is difficult to demarcate if the osteolytic lesions have no clearly boundary 16. For diminishing the measuring bias, the final results were averaged from the measurements by three researchers. Nevertheless, the manual segmentation is not simplified enough for clinicians. Second, this study was retrospective so that recall bias was existed. Additionally, the sample size was small in some groups. Thus, prospective studies and large sample size are necessary to provide more definitive scientific envidence.

## Conclusion

This is the first study about risk assessment of the severity and recurrence of MRONJ by analyzing the volumes of osteolytic lesions and measuring the radiodensity values of osteosclerotic lesions. MRONJ patients receiving intravenous bisphosphonates develop into large volumes of osteolytic lesions more easily. Long-term bisphosphonates duration is possibly related with higher bone density of osteosclerotic lesions, while higher density is not associated with the transformation from osteosclerotic lesions to osteolytic lesions. The high bone mineral density nearby post-surgical lesions is probably a predictor for MRONJ recurrence. Further prospective studies with large samle size are needed to provide more definitive envidence.

## Figures and Tables

**Table 1:** Volumetric analysis in osteolytic MRONJ lesions

Variables	N (%)	Volume (mm^3^) (mean ± SD)	P values
Bisphosphonates duration	-	3920.20±2967.65	0.094^#^
Age	-	3920.20±2967.65	0.578^#^
**Gender**			
Male	24 (45.3)	4763.75±3570.53	0.073
Female	29 (54.7)	3222.09±2183.78	
**Routes**			0.004^*^
Oral	8 (15.1)	2240.44±1200.13	
IV	45 (84.9)	4218.82±3094.32	
**Location**			0.519
Maxilla	18 (34.0)	4291.67±2676.13	
Mandible	35 (66.0)	3729.16±3126.97	
**Osteoporosis**			0.027^*^
No	39 (73.6)	4303.95±3294.91	
Yes	14 (26.4)	2851.18±1349.47	
**Chemotherapy**			0.159
No	25 (47.2)	3309.90±2448.62	
Yes	28 (52.8)	4465.11±3313.48	
**Targeted drugs**			0.408
No	37 (69.8)	3633.79±2222.82	
Yes	16 (30.2)	4582.52±4242.90	
**Immune suppressive drugs**			0.291
No	41 (77.4)	3685.11±3093.17	
Yes	12 (22.6)	4723.42±2435.41	

Notes: ^#^P values were calculated by general linear regression. *P values were statistically significant.

**Table 2:** Radiodensity values of osteosclerotic lesions in MRONJ patients

Variables	N (%)	Radiodensity values (mean ± SD)	P values
Bisphosphonates duration	25 (100)	896.25±162.06	0.040*^#^
Age	25 (100)	896.25±162.06	0.511^#^
**Gender**			
Male	13 (58.0)	856.11±179.56	0.276
Female	12 (52.0)	947.86±130.96	
**Routes**			NA
Oral	1 (4.00)	753.53	
IV	24 (96.0)	905.80±163.02	
**Location**			0.367
Maxilla	8 (32.0)	830.50±226.035	
Mandible	17 (68.0)	918.17±140.66	
**Osteoporosis**			0.403
No	20 (80.0)	909.57±168.50	
Yes	5 (20.0)	803.00±70.711	
**Chemotherapy**			0.777
No	13 (52.0)	908.22±88.630	
Yes	12 (48.0)	880.86±233.87	
**Targeted drugs**			0.888
No	16 (64.0)	891.60±169.37	
Yes	9 (36.0)	904.00±164.44	
**Immune suppressive drugs**			0.598
No	22 (88.0)	904.71±172.19	
Yes	3 (22.0)	837.00±22.627	

Notes: ^#^P values were calculated by liner regression. *P values were statistically significant.
